# 

**Published:** 1880-12

**Authors:** 


Pamphlets Received. An Obstetrical Case: Intra-Uterine Amputations. By Walter Coles, M.D., St Louis. Report from Transactions St. Louis Medical Society. September, 1880.

On the Relations of the Placenta to Post Partum Hemorrhages. By Walter Coles, M.D. Read before St. Louis Medical Society. March, 1880.

Department of the Interior, Bureau of Education. Medical Colleges in the United States.

Department of the Interior, Bureau of Education. Educational Transactions in France.

Seven Cases of Retroflexion of the Uterus, with Peritoncal Adhesions of the Fundus in the hollow of the Sacrum, treated by forcible separation of the adhesions. By Augustus F. Erich, M.D., Prof. of Diseases of Women in the College of Physicians and Surgeons, Baltimore, Md.

Puerperal Epilepsy and Protracted Gestation. By L. S. Oppenheimer, M.D., Demonstrator of Microscopy and Histology in the University of Louisville. Reprint from the American Practitioner. October, 1880.

Clinical Observations on the Radical Treatment of Fibroid Tumors of the Womb. By Wm. Goodell, M.D. Extracted from Transactions of the Medical Society of the State of Pennsylvania for 1880.

Motoris Pocket Series, No. 1. Diet for the Sick. By J. W. Holland, M.D.; Prof Materia Medica, Therapeutics, etc., in the University of Louisville. Price 25 cents.

